# Mechanotransduction at focal adhesions: from physiology to cancer development

**DOI:** 10.1111/jcmm.12045

**Published:** 2013-04-18

**Authors:** Jihye Seong, Ning Wang, Yingxiao Wang

**Affiliations:** a Neuroscience Program, University of IllinoisUrbana, IL, USA; b Department of Mechanical Science and Engineering, University of IllinoisUrbana, IL, USA; c Department of Bioengineering & Beckman Institute for Advanced Science and Technology, Department of Integrative and Molecular Physiology, Center for Biophysics and Computational Biology, Institute for Genomic Biology, University of IllinoisUrbana, IL, USA

**Keywords:** Mechanotransduction, Focal Adhesions, Extracellular Matrix, Integrin, Cancer, Invadopodia

## Abstract

Living cells are continuously exposed to mechanical cues, and can translate these signals into biochemical information (*e.g*. mechanotransduction). This process is crucial in many normal cellular functions, *e.g*. cell adhesion, migration, proliferation, and survival, as well as the progression of diseases such as cancer. Focal adhesions are the major sites of interactions between extracellular mechanical environments and intracellular biochemical signalling molecules/cytoskeleton, and hence focal adhesion proteins have been suggested to play important roles in mechanotransduction. Here, we overview the current molecular understanding in mechanotransduction occurring at focal adhesions. We also introduce recent studies on how extracellular matrix and mechanical microenvironments contribute to the development of cancer.

- Introduction
- Mechanical force at focal adhesions
  – Focal adhesions
  – Outside-in and inside-out force
- Mechanotransduction at focal adhesions
  – Fibronectin
  – Integrin α5β1
  – Talin
  – Vinculin
  – p130Cas
- Cancer development under mechanical microenvironment *via* focal adhesions
  – Matrix stiffening and tumour progression
  – tumour-repopulating cells selected by soft microenvironment
  – Podosomes, invadopodia and cancer metastasis
- Conclusions

## Introduction

Living cells receive various mechanical signals from their microenvironments. In particular, different types and density/network of extracellular matrix (ECM) molecules can provide different mechanical microenvironment of surface rigidity, cell anchoring points and topography. Because cells are physically connected to ECM microenvironment through the transmembrane receptor integrins at focal adhesions (FAs) 1, the outside mechanical signals can be sensed at these sites and translated into biochemical information through integrin-related signalling pathways 2, 3. Intracellular traction force, generated by actomyosin-derived contractility, can also be transferred to extracellular matrix through integrins at focal adhesions 4. Therefore, focal adhesions serve as crucial sites for both outside-in and inside-out mechanotransduction.

Recent discoveries unveiled the molecular mechanisms on how some of focal adhesion proteins can be activated by mechanical signals. For example, cellular traction force can stretch fibronectin to expose the sites required for its fibrillogenesis 5. Mechanical stretching can also unfold the talin rod domain, allowing the recruitment of vinculin to stabilize the connection between integrin and actin cytoskeleton 6. Vinculin can serve as a force-bearing molecule at focal adhesions and determine FA assembly and disassembly 7. In addition, mechanical stretching of p130Cas can increase its susceptibility to phosphorylation by Src family kinases 8. These examples share a general mechanism of mechanotransduction at focal adhesions, *i.e*. mechanical force can be translated as biochemical information by exposing the crucial sites of target molecules which are required for the downstream signalling pathways 9.

Mechanotransduction occurring at focal adhesions, in short term, can regulate cell spreading, shape and migration. In long term, it can also regulate gene expression, cell differentiation and the progression of diseases such as cancer 10. In this review, we focus on the molecular mechanisms on how mechanical force can be translated into biochemical signals at focal adhesions. We also introduce recent studies on how mechanical signals passing through focal adhesions contribute to the development of cancer.

## Mechanical force at focal adhesions

### Focal adhesions

**Fig. 1:**
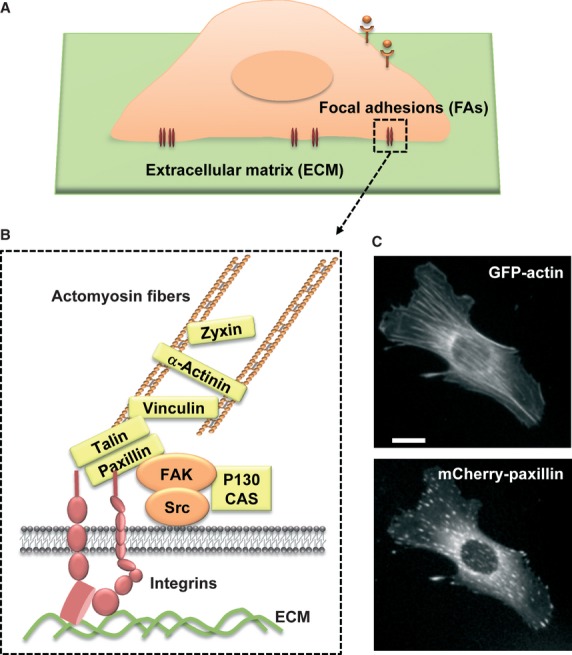
Focal adhesions. (**A**) Focal adhesions between a cell and extracellular matrix (ECM). (**B**) Structure and composition of focal adhesions. When bound to ECM (green), the transmembrane integrin receptor (red) can recruit signalling proteins (orange) and structural proteins (yellow), linking actomyosin fibres. (**C**) Actin stress fibres can be developed from focal adhesions, as visualized by GFP-actin and mCherry-paxillin staining in a mouse embryonic fibroblast cell. Scale bar = 10 μm.

Focal adhesions are the contact sites of cells to outside extracellular matrix through transmembrane proteins integrins (Fig. 1A) 1. Integrins are heterodimeric receptors containing α and β subunits, and so far 24 different subtypes of integrins have been identified in vertebrates with the combination of 18 α and 8 β subunits. These integrin subtypes allow the diverse and specific recognition of various ECM proteins, *e.g*. fibronectin, fibrinogen, collagen, vitronectin and laminin 11.

After the ligation of integrins with ECM proteins, many structural and signalling proteins are recruited to focal adhesions (Fig. 1B). Signalling proteins at focal adhesions include kinases *e.g*. Src, focal adhesion kinase (FAK), integrin-linked kinase (ILK), and phosphatase *e.g*. receptor-like tyrosine phosphatase α (RPTP-α). As integrin itself lacks the enzymatic activity, these signalling proteins at focal adhesions are crucial to transfer extracellular mechanical information inside the cells. For example, integrin-mediated activation of Src and FAK can regulate Rho GTPases, which then regulate the organization of actin cytoskeleton 12, 13.

Structural proteins, *e.g*. talin, paxillin, vinculin and zyxin, link other focal adhesion proteins and actin cytoskeleton (Fig. 1). For example, ECM-bound integrins can recruit an adaptor molecule talin to focal adhesions. Talin has the binding site for another molecule vinculin, which then binds to actin cytoskeleton 14, 15. As such, outside ECM can be physically connected to intracellular cytoskeleton through integrin and focal adhesion proteins. Therefore, owing to their physical location at the interface between extracellular microenvironments and intracellular space, focal adhesions play crucial roles in sensing mechanical signals both from outside and inside 2, 3.

### Outside-in and inside-out force

Extracellular mechanical cues, *e.g*. substrate stiffness 16–22 and shear stress 23–25, control many cellular processes. These outside forces can be transmitted through focal adhesions by regulating the organization and/or binding strength of integrins to ECM protein or integrin. For example, mechanical forces can cause the allosteric change in extracellular domains of integrin αvβ3, increasing the bond strength between integrin αvβ3 and fibronectin 26. Subsequently, more signalling proteins such as Src and FAK can be recruited to integrins, which then regulate the dynamics of actin cytoskeleton through RhoA GTPases. At more stable focal adhesions, stronger actin stress fibres can also be developed. As such, the force-induced conformation changes in integrin can regulate focal adhesion dynamics and the organization of actin cytoskeleton, which regulate cell adhesion, shape and migration 12. It has been also well reported that extracellular mechanical stimuli can activate PI3K 27 and MAP kinase including ERK and JNK 28, 29. For example, stiffened matrix induced the activation of PI3K through integrin signalling 27, and shear stress-induced activation of FAK and its rapid association with Grb2 can link to the MAP kinase signalling 28. These signalling molecules, which are involved in important cellular processes *e.g*. proliferation and survival, are often deregulated in cancer 30, 31. Therefore, outside mechanical signals can be sensed through focal adhesions to control intracellular physiological processes as well as cancer development.

Intracellular mechanical signals can also be transferred to extracellular microenvironments *via* focal adhesions 32, 33. Actin cytoskeleton is linked to ECM-bound integrins at focal adhesions. While one end of actin fibres is connected to integrins, the other end grows toward inside the cell by its retrograde flow, developing tensile force. This actomyosin-derived intracellular tensile force can affect the assembly of extracellular matrix through focal adhesions 4. For example, it has been suggested that integrin-bound ECM protein fibronectin can be stretched by the actomyosin-derived tensile force. This conformational change in fibronectin can expose crucial sites for its fibrillogenesis 34. The tensioned state of integrin α5β1, supported by actomyosin-derived force can increase the bond strength between integrin and fibronectin 35. Myosin IIB-mediated intracellular tensile force can be also transmitted to collagen fibres through integrin α2β1, resulting in the transport of collagen fibres and the contraction of *in vitro* 3D collagen matrix 36. Thus, intracellular mechanical signals can be sensed at focal adhesions to control extracellular environment. As such, bidirectional mechanotransduction, *i.e*. outside-in and inside-out, can be transmitted through integrins at focal adhesions to control intracellular signalling or extracellular ECM matrix. These mechanotransduction processes should be tightly regulated to maintain the homeostasis of the cell 10, 37.

## Mechanotransduction at focal adhesions

Mechanical force can be translated to biochemical signals when the force can cause the conformational changes in the molecules to expose the otherwise masked sites, which are crucial for the further enzymatic activity or molecular recognition 9. In particular, focal adhesion proteins may be subjected to mechanical stretching by actomyosin-derived tensile force exerted on the ECM-bound integrins. In this section, we will overview the molecular mechanisms on how mechanical force regulates the molecular activity of focal adhesion proteins 5–8.

### Fibronectin

Fibronectin, a major component of extracellular matrix, is a multimodular protein containing type I, II and III repeating units. Whereas type I and II modules are stabilized by intramodular sulphide bonds, type III modules are mechanically flexible and can be extended by forces at physiological level 9. The type III domain of fibronectin has a seven-stranded β-barrel structure with the twisted β-strands in the opposing β-sheets. Unravelling of the type III module can be initiated by the alignment of these twisted β-strands along the force vector. The alignment can be achieved by the disruption of backbone hydrogen bonds between β-strands and the subsequent penetration of water molecule 38. This can cause the exposure of A- and B-strands to induce the assembly of fibronectin matrix 39. Thus, fibronectin type III modules can be extended by mechanical force, which will expose the cryptic sites crucial for fibrillogenesis of fibronectin. In fact, utilizing fluorescence resonance energy transfer (FRET) technology, it has been experimentally shown that a compact structure of fibronectin is unfolded in fibronectin fibrils by cellular traction force 5.

### Integrin α5β1

Intracellular traction force, generated by actomyosin-mediated contractility, can control the bond strength between fibronectin and integrin, and regulate intracellular signalling pathways such as FAK activation 35. Fibronectin primarily binds to integrin α5β1 through its type III_9-10_ modules. While the RGD (Arg-Gly-Asp) peptide in the type III_9_ domain of fibronectin binds to the extracellular domain of integrin β1, the synergy site in the type III_10_ domain of fibronectin is normally buried but can be exposed to integrin α5, only when the integrin α5β1 becomes tensioned by actomyosin-mediated contractility. In particular, Arg1374 and Arg1379 were critical for this additional binding to integrin α5. FAK phosphorylation at Tyr397 was observed only when the tensioned integrin α5β1 binds to both type III_9_ and III_10_ modules of fibronectin. Thus, intracellular traction force can switch the conformation of integrins to increase the bond strength to ECM, which in turn, regulates intracellular signalling pathways.

### Talin

Talin is a structural molecule at focal adhesions, which link integrins to actin cytoskeleton 14. Talin is composed of a globular head and a long rod domain. The FERM (four-point one, ezrin, radixin, moesin) F3 domain in the talin head provides the binding sites for the cytoplasmic part of integrin β subunit 40. The talin rod contains several binding sites for vinculin, which then connects to actin cytoskeleton. The vinculin-binding sites (VBSs), spanning residues 482–889 in the talin rod, are in the α-helix bundle region, which consists of 5-helix bundles surrounded by another neighbouring 7-helix bundle 15. Vinculin can interact with the hydrophobic residues on one side of α-helix in the talin rod. Some of those residues, for example, the VBS1 in the helix 4, are normally buried within the hydrophobic cores of helical bundles, contributing the stability of the folded structure 41. Thus, researchers have been suggested that mechanical force may destabilize the helical bundles in the talin rod, which then expose the cryptic vinculin-binding sites to recruit vinculin to focal adhesions. Consistently, it has been shown that both external and internal forces can increase the recruitment of vinculin at focal adhesion complexes 42, 43.

This hypothesis was further experimentally demonstrated *in vitro*, at the single-molecular level, by del Rio and colleagues 6. When the force was applied (up to 12 pN) to the helical bundles of the talin rod molecule utilizing magnetic tweezers, the force-dependent increase in vinculin binding to the talin rod domain was observed, by detecting the fluorescence photobleaching events of Alexa 488-labelled vinculin. In the absence of force, one binding event was observed indicating the existence of one readily exposed vinculin-binding site in the talin rod, most likely in the helix 9 as predicted in a previous study 44. The application of force increased the binding event from one to three, suggesting that two cryptic VBSs may be exposed by force-induced stretching of talin rod domain. In addition, a sequential unfolding pattern of the talin rod molecule was observed by single-molecule force extension spectroscopy, indicating different mechanical stabilities within the molecule. It was also predicted by molecular dynamic calculations that the applied force can sequentially break the helical bundles region (helix 1–12) of the talin rod into helix 1–8 and helix 9–12, and then helix 1–8 into helix 1–5 and helix 6–8. These three mechanical intermediate of talin rod domain can explain the three binding events explained above. Therefore, these studies suggest that the stretching of single talin rod molecule, possibly by actomyosin-derived tensile force in cells, can recruit vinculin by exposing its cryptic binding sites, which can strengthen the connections between integrin and actin cytoskeleton. It remains unclear, however, what the threshold force is *in vivo* to unfold the talin molecule and facilitate the vinculin binding.

### Vinculin

Vinculin, which can be recruited to focal adhesions by force, can be also stretched by intracellular tensile force as a force-bearing molecule 7. Vinculin is composed of a head domain (Vh) and a tail domain (Vt) which are connected by a flexible linker. Because Vh binds to talin and Vt interacts with F-actin, vinculin can be localized at focal adhesions between integrins and actomyosin fibres. To measure the tensile force transmitted by the vinculin molecule in living cells, Grashoff *et al*. 7 created a vinculin tension sensor based on fluorescence resonance energy transfer (FRET). Vinculin tension sensor contains Vh and Vt connected by a sensor module, which is composed of an elastic domain between FRET pair fluorescence proteins, mTFP1 and Venus. As tension is applied to the vinculin tension sensor, FRET efficiency will decrease because of the increased distance between two fluorescence proteins. Thus, this biosensor allowed a direct measurement of tensile force in living cells.

Utilizing the vinculin tension sensor, Grashoff *et al*. revealed that vinculin is under high tension at the newly assembled focal adhesions in the leading edges of the migrating cells. The force across the vinculin was low at the sliding or disassembling FAs at the retracting edges. These data suggest that vinculin bears the tensile force stabilizing FAs, and when vinculin fails to bear force under tension, the FAs will disassembly. Therefore, vinculin can serve as a mechanosensor under tension to determine either FA assembly or disassembly.

### p130Cas

A substrate of Src kinase, p130Cas (Crk-associated substrate), has also been suggested as a mechanotransducer 8. p130Cas contains N-terminal SH3 domain, Cas substrate domain (CasSD) and C-terminal Src binding sequence YDYV. CasSD containing 15 repeats of YXXP motif 45 can be phosphorylated by Src kinase. p130Cas is localized at focal adhesions through the interactions with the FAK proline-rich region and Src SH2 domain *via* its N-terminal SH3 domain and the C-terminal Src binding sequence, respectively 46. Additional FA targeting site was also identified as a helix-loop-helix motif at the extreme C-terminus of p130Cas 47. As the FAT domain of FAK is associated with talin, which then interacts with actin cytoskeleton, p130Cas can be subjected to actomyosin-generated force at focal adhesions. In fact, it has been reported that, in detergent-insoluble cytoskeleton complexes, cell stretching can increase the phosphorylation of CasSD by Src family kinases (SFKs) and activate a small GTPase Rap1 in a force-dependent manner 48.

Sawada *et al*. designed an *in vitro* system to test whether the CasSD can be extended by mechanical stretching, and whether this extension can cause the increased phosphorylation of CasSD by SFK 8. In this system, the biotinylated CasSD on both N- and C-termini was bound to an avidin-coated latex substrate. Amino- and carboxy-terminal regions of yellow fluorescence protein (YFP-N and YFP-C) were further fused to each end of NC-biotinylated CasSD. The extension of CasSD can separate two halves of YFP, which allows the binding of the exogenous YFP-N containing His-tag. Thus, when the latex substrate is stretched, the extension of CasSD can be confirmed by both loss of yellow fluorescence and by the detection of Hig-tag binding to the biotinylated CasSD.

Src family kinases were applied in this system and the CasSD phosphorylation level with or without the mechanical stretching was monitored. The stretching-induced CasSD extension resulted in an enhanced phosphorylation of CasSD by SFK without change in their kinase activity. These data indicate that the extension of Cas substrate domains increases its susceptibility to phosphorylation by SFK, which can activate further downstream signalling pathways. The detailed structure of CasSD is currently not clear, but the stretching-induced extension of CasSD may expose cryptic sites for the phosphorylation by SFK. Therefore, p130Cas can function as a mechanical sensor by priming its phosphorylation.

## Cancer development under mechanical microenvironment *via* focal adhesions

### Matrix stiffening and tumour progression

As discussed in the previous sections, cells continuously receive the mechanical signals from extracellular microenvironment through focal adhesions. Cells respond to these signals through integrin-related signalling pathways to maintain proper cellular functions in cell shape, migration and survival. Thus, the loss of the tension regulation by changes in the extracellular microenvironment can lead to the cancer progression and metastasis 10, 49, 50. In fact, changes in ECM microenvironments induced the breast cancer progression 27, 51, and conversely, malignant carcinoma cell can become normal by changing their microenvironment 52. Therefore, microenvironments play crucial roles in cell homeostasis as well as cancer development.

Collagen is one of major ECM proteins in the extracellular microenvironment, and it has been reported that the turnover of collagen by matrix metalloproteinase (MMP) is enhanced during cancer development 53, 54. MMP collagenases can cleave fibrillar collagen into fragments, creating gaps in the extracellular matrix for cancer cells to migrate out for cancer metastasis. The matrix degradation can occur at focal adhesions in many cancer cell types, and the targeting of MT1-MMP to these sites is regulated by the FAK-p130Cas complex as well as the Src-induced phosphorylation at Tyr 573 of MT1-MMP 55. However, MMP inhibitors failed in the clinical trials to prevent cancer development 56. In fact, it has been also reported that collagen expression and deposition are increased during cancer development. More importantly, collagen cross-linking can cause tissue fibrosis 57, which may be involved in tumour progression, as tumours are generally stiffer than normal tissue.

Levental *et al*. presented that the collagen cross-linking by lysyl oxidase, which can stiffen the matrix, can cause breast tumourigenesis through integrin signalling pathways 27. Because collagen as an ECM protein can interact with integrins, the changes in ECM rigidity can be translated inside cells through integrins at focal adhesions. In fact, the authors showed that enhanced integrin signalling in the stiffened matrix, *e.g*. focal adhesions formation and increased PI3K activity 58, 59, promotes the cell invasion and breast cancer malignancy. The inhibition of collagen cross-linking or integrin signalling reduced cell invasion and tumour progression. Interestingly, stiffened ECM alone was not sufficient to induce mammary tissue invasion, but the additional oncogenic signalling was required, such as ErbB2 or TGF-β 60, suggesting that the coordination of both mechanical and biochemical factors are crucial to promote the tumour progression. Therefore, stiff mechanical microenvironment can promote tumourigenesis through integrin-dependent mechanotransduction at focal adhesions.

### Tumour-repopulating cells selected by soft microenvironment

It has been shown that stem cell–like cancer cells, which comprise a small fraction of total cancer cell populations, can generate tumours when implanted in animal hosts, suggesting their crucial roles in the relapses of cancer 61, 62. Stem cell–like cancer cells are self-renewing and can be differentiated into non-stem cells, thus tumours are composed of heterogeneous cancer cells with different stages of differentiation 63. Liu and colleagues showed, in a recent report, that this tumour-repopulating cell (TRC) population can be selected by soft mechanical microenvironment 64. They isolated TRCs from B16-F1 melanoma cells by culturing them in soft 3D fibrin gels. TRCs express several key stem cell markers for self-renewal, *e.g*. Sox-2, and as few as 10 TRCs were able to form a tumour when seeded in mice.

Tumour-repopulating cells proliferate better in the softer gels than stiff gels, suggesting that the soft mechanical microenvironment can contribute to the selection of this tumourigenic cell population and thus tumour progression. The selection of TRCs was more efficient in 3D gels than on 2D gels of similar stiffness coated with fibrin. The size of the tumour spheroids was smaller in the 3D collagen-I gel and their proliferating rate was also slow, suggesting that the specific integrin subtypes, *e.g*. integrin αvβ3 interacting with fibrin, and/or ECM organizations may be involved in the transduction of mechanical signals through focal adhesions and furthermore in the selection of TRCs. Therefore, specific microenvironment of soft 3D fibrin gels promotes the proliferation of this tumourigenic cell population. Interestingly, TRCs keep their softness even in the rigid microenvironment, which may allow the efficient penetration into different tissues. As they can exert stronger traction in the stiff gels, soft TRCs can migrate into the rigid ECM. Therefore, TRCs can be highly tumourigenic with these unique biomechanical features in addition to their survival and high proliferation rate in the soft microenvironment.

The discrepancy between this interesting finding of TRCs proliferation in the soft microenvironment and the previous report that the stiffened matrix promotes tumour progression 27 implies that the complex mechanisms involved in the cancer development under mechanical microenvironment. Cancer cells which invade through stiffened matrix may be mostly differentiated and only contain a small population of undifferentiated cancer cells, as the stem cell–like TRCs can survive better in the soft microenvironment. However, after intravasation and extravasation, it is possible that this small portion of TRCs can eventually survive and proliferate to form a tumour in other tissues, resulting in cancer metastasis 65, 66. Therefore, mechanical cues, transmitted through focal adhesions, play crucial roles in different steps of cancer development.

### Podosomes, invadopodia and cancer metastasis

Podosomes and invadopodia are dynamic cell-matrix contact structures often found in invasive normal and cancer cells. Podosomes/invadopodia share many molecules with focal adhesions, but they are more dynamic and have the unique structure which is composed of actin-rich core surrounded by integrin-associated proteins such as paxillin and talin 67, 68. It is interesting that the highly dynamic podosome structures, which may not be stable enough to transmit sustained forces for long periods of time (>10 min.), are also mechanosensors like focal adhesions 69. In this study, Collin *et al*. found that myosin II, localized just outside and at the podosome actin ring, is essential for the structure and dynamics of podosomes 69. They showed that the myosin-dependent endogenous forces can be transmitted to outside microenvironment, through podosomes, to exert strong tractions on the substrate. In turn, mechanical signals from microenvironment, *e.g*. substrate rigidity, were transferred to inside cells through podosomes, as the traction exerted by podosomes was sensitive to the substrate rigidity. Podosomes also sense direct mechanical stresses which are applied to the apical cell surface by magnetic twisting cytometry. The deformations in the podosomes were sensitive to the magnitude of the applied force. These results suggest that podosomes can function as mechanosensors transmitting both inside-out and outside-in forces.

Recent study by Hoshino *et al*. showed that the deregulated high PI3K activity with low PKCα signalling in cancer cells can facilitate the transition from focal adhesions to invadopodia 70. Invadipodia are involved in matrix degradation, more actively than focal adhesions, and thus cancer invasion/metastasis 71, 72. These dynamic structures can actively sense and respond to the mechanical signals in the caner microenvironment 69, which may also contribute to the cancer metastasis.

## Conclusions

External mechanical cues can be transmitted through focal adhesions to regulate cellular processes. Intracellular traction force can modify extracellular environment through focal adhesions. Therefore, focal adhesions function as the important sites of the bidirectional mechanotransduction. Accordingly, many focal adhesion proteins are suggested as mechanotransducers, *e.g*. fibronectin, integrin, talin, vinculin and p130Cas. Mechanotransduction at focal adhesions, where actomyosin-tensile force is applied on ECM-bound integrins, uses a general molecular mechanism in that mechanical stretching can unfold or extend the mechanotransducer to expose crucial sites for the induction of molecular interactions or activities. While the tight regulation of mechanotransduction at focal adhesions is required to maintain tensional homeostasis of the cell, both soft and rigid microenvironments contribute to the different steps of cancer development. Therefore, correct understanding of mechanotransduction at focal adhesions will provide important insights on cellular processes as well as cancer development.
